# Equity in aid allocation and distribution: A qualitative study of key stakeholders in Northern Uganda

**DOI:** 10.1371/journal.pone.0226612

**Published:** 2019-12-16

**Authors:** Primus Che Chi, Patience Bulage, Gudrun Østby

**Affiliations:** 1 Department of Health Systems and Research Ethics, KEMRI-Wellcome Trust Research Programme, Kilifi, Kenya; 2 Peace Research Institute Oslo (PRIO), Oslo, Norway; 3 Health Economics and HIV/AIDS Research Division (HEARD), University of KwaZulu-Natal, Durban, South Africa; Newcastle University, UNITED KINGDOM

## Abstract

The Sustainable Development Goals have spurred a growing interest in and focus on equitable development. In theory, donors can play an important role in promoting equity within a country by providing services, influencing government policies and incorporating equity into decision-making. However, we know little about whether this actually happens on the ground. We conduct what we believe is the first study to explore the extent to which equity is prioritised in the allocation and distribution of aid, based on in-depth interviews with government officials, bilateral and international donors, and implementing partners operating in Northern Uganda. We find that a broad category of people are perceived to be marginalised/vulnerable, with a substantial segment largely untargeted by major donor programmes. Various stakeholders employ a wide range of strategies to identify the most vulnerable individuals and groups, including the use of available data and statistics, consultation and engagement with relevant stakeholders, and undertaking primary data collection. The strategies used to incorporate equity in aid allocation and distribution include: targeting the regions of Northern Uganda and Karamoja in particular, targeting both refugees and host populations in refugee-hosting districts, prioritising the critically vulnerable in any aid distribution process, and using specific tools and consultants to ensure that major equity issues are addressed in proposals. Challenges undermining the process include poor understanding of the concept of equity among some implementing partners, lack of comprehensively disaggregated data, corruption, and political interference in choice of aid location from government officials and donors.

## Background

Sustainable Development Goal (SDG) number 10 focuses on reducing inequalities within and among countries, with one of the targets being to “encourage official development assistance and financial flows, including foreign direct investment, to states where the need is greatest, in particular least developed countries, African countries, … in accordance with their national plans and programmes” [1]. This target re-affirms the potential importance of development assistance or aid in bridging inequalities between and within countries. The launch of the SDGs has therefore spurred a growing interest in and focus on equitable development, with equity increasingly considered as a central aspect to development programming. The World Health Organization defines equity as the absence of avoidable or remediable differences among groups of people, whether those groups are defined socially, economically, demographically, or geographically [2]. Equitable development is therefore defined as is an approach for meeting the needs of underserved communities through policies and programmes that reduce disparities while fostering places that are healthy and vibrant [3]. It hence involves a deliberate and intentional effort to reduce disparities within and across countries in the development process. Equitable development is an integral component of the global sustainable development agenda that advocates for development that meets the needs of the present, without compromising the ability of future generations to meet their own needs. For sustainable development to be attained the United Nations advocates that there “must be promotion of sustainable, inclusive and equitable economic growth, creating greater opportunities for all, reducing inequalities, raising basic standards of living, fostering *equitable social development* and inclusion, and promoting integrated and sustainable management of natural resources and ecosystems” [4].

Development assistance is a complex and multi-faceted field with the goal of improving the lives of the world’s poorest people. Its activities can range from one-off projects to multi-year global programmes targeting scourges like HIV/AIDS to emergency relief, like the instant rescue efforts that follow disasters such as the 2004 Asian Tsunami or the 2010 Haiti earthquake [5]. These represent a small percentage of the developing world’s challenges. In reality however, the bulk of development co-operation or assistance involves governments, non-governmental organisations (NGOs) and is planned out well in advance. It often has long-term goals, such as improving access to healthcare and education, building infrastructure, or reinforcing countries’ capacity to run their own affairs. To achieve these goals, different approaches are taken, including providing grants and loans to developing countries; supplying experts, equipment and training; providing funds to the governments of developing countries directly or bypassing them altogether to build projects on the ground, and so on. The range of actors, too, is diverse, ranging from the donor governments in developing and wealthy countries, the international agencies answerable to multiple governments, like the World Bank, the United Nations, the Asian Development Bank or the OECD, the civil society organisations, the religious charities and private foundations, like the Gates Foundation that today plays a bigger role than many governments. And, of course, there are the developing countries themselves and, within them, their own governments, and agencies. At this point, it is important to highlight that beyond the often mentioned goal of improving economic growth and development and reducing inequalities within the aid recipient country, studies have found that donors can equally use their development assistance to enhance their trade interests within the recipient country, and also promoting the donor country’s long-term security [6, 7]. The primary motive of any donor development assistance is therefore not always easy to decipher.

Although development assistance can play an important role in equitable development between and within countries, the extent to which the major actors involved in the allocation and distribution of development aid prioritise this in policy and practice is unclear. The literature on development aid and equity is quite sparse. As such, the background/literature review for this paper is largely drawn from the sole paper that has arguably delved deeply into this issue; “*Equity in development*: *Why it is important and how to achieve it*” by Jones [8] that was published in 2009. The review found that equity is under-represented in development policy and practice, due to poor understanding of the concept and its implications [8]. Although the concept is quite a visible commitment for most donors and appears to be incorporated in their policies and programming, it tends to be used in a rather vague fashion [9]. Considering that different actors may have a different understanding of the concept of equity, this may have a direct effect on their policies and practices towards improving equity. For example, equity can be viewed as distributive justice, which focuses on equitably allocating resources with the aim of reducing disparities, inequities and improving outcomes [10]. In the context of allocating and distributing of aid, Jones has defined equity as the distribution of aid based on the *needs* of the people, i.e. proportional to the extent to which they are missing them [8]. Based on this understanding of equity, the promotion of equity in the allocation and distribution of development assistance by donors and implementing organizations will contribute to equitable and sustainable development by reducing poverty, and economic disparities. The concept of equity can be understood through three principles; equal life chances, equal concern for people’s needs and meritocracy, as described in Table 1 [8].

**Table 1:** Principles of equity.

Principle of equity	Description
Equal life chances	There should be no differences in outcome based on factors for which people cannot be held responsible
Equal concern for people’s needs	Some goods/services are matters of necessity and should be distributed proportional to people’s level of need and nothing else
Meritocracy	Positions in society and rewards should be distributed to reflect differences in effort and ability, based on fair competition

Source: [8].

However, within the aid literature, the predominant discourses on aid and equity have focused on the first two principles of equal life chances and equal concern for people’s needs. Arguably the third principle of meritocracy is best achieved after the previous two have been attained as prevailing inequalities in life chances and concerns for people’s needs may create a situation of unfair competition among individuals in the same community, region or country. For example, prioritizing the principle of meritocracy in employment when inequalities in access to education prevail, would only further exacerbate the prevailing inequities. As such, most of the efforts to address inequities within countries, especially in the area of aid allocation and distribution are understandably focused on ensuring equal life chances and equal concern for people’s needs. For example, in the domain of health, equity can be attained through the principle of social justice, which aims to improve population health by levelling access and opportunity to resources for achieving good health, especially among vulnerable populations, with the goal of reducing disparities in health status [10].

Donors and international non-government organisations (NGOs) can play a crucial role in promoting equity within a country. Jones [8] has proposed at least three different roles that donors and other development actors can play in promoting equity within a country: providing services; influencing government policies; and incorporating equity into decision making. Donors can provide services by using aid allocation and distribution policies that target disadvantaged/marginalised groups, using transfers to help protect people from falling below a specific minimum level of wellbeing, and contributing towards shifting of existing power structures. This can be achieved through the provision of basic health and education services targeting the worst off in a society; and implementing programmes and projects that target assistance towards marginalised and vulnerable groups of the society. With respect to influencing government policies, donors can use a number of strategies to influence aid recipient governments to design and implement pro-equity policies. This is largely hinged on the notion of neutrality since development partners are separate from the national powers that may reinforce social, political and economic inequalities [11]. Donor strategies to influence government policies may range from using ‘carrot and stick’ approaches, creating incentives for institutional changes and tying the costs to access to new loans or markets. Finally, donors can incorporate equity into their decision making by more consistently and coherently taking equity as a goal for their work, incorporating an understanding of the pressing inequities within countries and informing assessment of the potential ramifications for equity of their work. Examples of incorporating equity in decision making could include undertaking aid allocations and distribution based on data that is disaggregated on various social, political and cultural categories; and incorporating weightings based on the levels of needs of the recipients in the allocation and distribution of aid. While these are not an exhaustive list of potential roles that donors can play in promoting equity within a country, the extent to which donors could play these roles might vary from one context to another. However, what these strategies highlight is that, given the increasing interdependence between the donors and recipient nations, donors can rely on this relationship to strategically promote equitable development [11].

To date, the most extensive work on development assistance and equity has been the review by Jones highlighted above. Building on this work, there is the need to explore how the concept of equity is perceived among key development stakeholders (i.e. what equity in aid allocation and distribution means to different stakeholders), how the perceived meaning or understanding of equity is translated or operationalised in practice during aid allocation and distribution, and the inherent challenges associated operationalising the various perceptions of equity in aid allocation and distribution. These are some of the main contributions of this study to the current development aid and equity literature, especially the work by Jones [8]. To achieve this, a case study of an aid recipient country was undertaken. Our goal is to move beyond the previous reviews to actual engagement with key actors on the ground within an aid recipient country to have a deeper and current understanding of operationalising equity in aid allocation and distribution. The aim is moving beyond what ought to be done to what is being done on the ground.

Uganda has been a major recipient of donor aid for the past decades and was regarded as one of the donor darlings on the African continent. For example, between 2003 and 2012 Uganda received more than $16 billion in official development assistance, ranking them as the 13th largest recipient worldwide. Between 2006 and 2010, about 87% of all of Uganda’s bilateral aid came from eight bilateral donors; USA, UK, Sweden, Norway, Denmark, Ireland, Germany and the Netherlands, while over 90% of the multilateral aid was provided by only five international organisations (World Bank, European Union, Africa Development Bank, Global Fund and UNICEF) [12]. Although aid is perceived to have played an important role in poverty reduction in the country over the past decades, it is however unclear how various donors, the government and implementing partners promote equity in the allocation and distribution of aid within the country.

## Aim and objectives

### Aim

This study seeks to understand the extent to which equity is incorporated into the allocation and distribution of development aid in Uganda by major stakeholders (donors, recipient government, and implementing organisations), with a focus on Northern Uganda.

### Objectives

1. To explore the prevailing strategies for identifying and reaching the most vulnerable groups
2. To identify the strategies to incorporate equity in the allocation and distribution of aid
3. To document the challenges/barriers to incorporating equity in the allocation and distribution of aid

## Materials and methods

### Study setting

Fieldwork was undertaken in Uganda, specifically in the towns of Gulu, in Northern Uganda and Kampala, the country’s capital between May and June 2017. Gulu is the commercial and administrative headquarters of Northern Uganda and was one of the districts that was affected by the 20 years (1986 to 2006) armed conflict between Uganda government and Joseph Kony’s Lord’s Resistance Army rebels.

Due to the previous prolonged war in Northern Uganda, the region was economically and socially cut off from the rest of Uganda and left behind in terms of development and provision of necessary services [13]. Nationally, there is a large and increasing variations in poverty, with the Northern region having the largest proportion of the poor compared to other regions of the country [14]. For example, in 2006 the proportion of the poor in Northern Uganda stood at 39% compared to 29%, 17% and 15% for the Eastern, Western and Central regions respectively. By 2013 the share of the poor in Northern Uganda had risen to 47% compared to 37%, 10%, and 6% for the Eastern, Western and Central regions respectively. During this period, the annual percentage reduction in poverty across the Western and Central regions are about two times higher compared to that in Northern Uganda. However, in recent years following the gradual return of peace in Northern Uganda, economic growth and poverty reduction (as defined by the World Bank’s extreme poverty threshold of US$1.90 a day) has been experienced, a situation partly accounted for by increased agricultural productivity, stable and favourable prices for agricultural produce locally and internationally, and increased urbanization [13]. Additionally, following the end of the conflict in 2006, substantial development aid has been channelled to the region. The choice of Gulu was based on its conflict history and the fact that it had received substantial donor aid as a result of the conflict. For example, during the by 2010 (the early years of the post-conflict period), the number of NGOs/ implementing organizations operating in Gulu was as high as 120 [15].

### Study participants

The study participants included major donors in Uganda, government officials and donor aid-implementing nongovernmental/ civil society organizations operating in Northern Uganda. The choice of participants was based on their role, experience and/or knowledge in the allocation, distribution, and/or coordination of development assistance. Participants from the government were senior bureaucrats at the national and district levels, those from the implementing organizations were also senior-level managerial/ administrative staff, including executive directors, executive secretaries, head of departments/ units among others at the regional, district, and sub-district levels; and those of the donor agencies were mostly heads or senior level technical staff within specific sections/ units. The participants were therefore knowledgeable of the issues under discussion. All representatives from the donor agencies and national level government officials were based in Kampala while the NGO participants and local level government officials were based in Gulu, Northern Uganda.

A total of 25 interviews were conducted, including 22 individual and three group interviews, involving a total of 33 persons. Of the interviews 25 interviews, 3, 8 and 14 were with government officials, international donors and implementing partners (non-governmental organisations) respectively, as shown on Table 2.

**Table 2:** List and categories of participating institutions.

Participant categories		
Donors (8)	Government Officials (3)	Implementing partners (14)
SIDA NORAD DANIDA (2 interviews) USAID DFID EU (group interview of 2) World Bank (group interview of 2)	Ministry of Finance (2 interviews) District Health Office	Save the Children Comboni Samaritans (group interview of 3) Action Aid Gulu NGO Forum Flama Uganda Reproductive Health Uganda (RHU) Gulu Women with Disabilities Union (GUWODU) (group interview of 5) Straight Talk Caritas Aid Africa Rural Focus Volunteer Action Network (VacNet) Gulu Women's Economic Development and Globalization (GWED-G) Charity for Peace Foundation (CPF)

### Data collection methods

Data was collected through face-to-face in-depth individual and group interviews (II and GI), using interview guides. Group interviews involved two—five participants from the same organization. The study was initially designed as an individual interview per organization. However, in some of the organizations the main staff who accepted our invitation to participate in the study invited one—four more co-workers to join in the interview. The participants for each group interview tended to fill in for each other and provided additional perspectives on the issues discussed. All interviews were conducted at the offices of the various participating institutions/ organizations. Interviews lasted from 30–90 minutes and were conducted by the first author (PCC), occasionally with support one or two research assistants who also served as notetakers. The interview guide for the entire study is available in the ‘Supporting Information’ section (Additional File 1).

### Ethical considerations

The proposal was submitted to the Gulu University Institutional Review Board (the ethics committee serving our main study site—Gulu district) for review and approval prior to commencement of the study. Written informed consent was obtained from all study participants, including consent for audio-recording of the interviews. Where consent for audio-recording was not obtained, detailed notetaking was done. We sought to protect participants privacy and confidentiality by reporting the study findings, especially the quotes anonymously and without mentioning the specific institution that participants represented and ensuring that only members of the research team had access to the audio recordings and unredacted transcripts. All relevant permits and approvals were obtained prior to conducting the study.

### Data collection and management

The majority of the interviews were audio-recorded and later subjected to verbatim transcription. In two of the interviews with donors, permission to use audio-recorders was not granted and notes were taken by two researcher assistants during the interviews. All the interviews were undertaken in the English language. Transcripts were later managed using Nvivo and MS Word. Analyses was by thematic analysis using the framework approach [16] as described elsewhere [17]. Briefly, after familiarisation with the interview transcripts, three of the transcripts were deductively and inductively coded. The codes were then developed into an analytical framework and the rest of the transcripts were coded by applying the developed analytical framework. The data was further summarised by category from each transcript (charting) and the final stage involved interpretation of the data. Data collection was undertaken from June—July 2017.

## Results

The results are presented in three sub-sections. In sub-section I, the strategies utilised by participants to identify the most marginalised and vulnerable, including their perception of which groups are marginalised/vulnerable are presented. Sub-section II outlines participants’ perceptions of what equity in aid allocation and distribution entails, and how this is operationalised in the process of aid allocation and distribution. In sub-section III, the challenges experienced by the participants in incorporating equity in aid allocation and distribution is presented. Quotes from donors, implementation partners and government officials are designated as *Donor*, *IP*, and *Gov’t* respectively. Individual interviews are designated as *II* while group interviews are designated as *GI*.

### *I*. Strategies for identifying the most marginalised/ vulnerable

#### A. Marginalised/vulnerable people: who are they?

To explore strategies for identifying the most vulnerable/marginalised groups, participants were first prompted to identify those they categorise as marginalised/vulnerable, and what made them vulnerable. Most of the respondents indicated that their categorisation of vulnerability was largely based on socio-economic status and coping capacity. Based on these, the respondents identified a broad category of individuals and groups as marginalised or vulnerable. We classified them into two major categories; the *mainstream* marginalised or vulnerable groups, and ‘*forgotten*’ marginalised or vulnerable groups. The mainstream marginalised groups included those that are broadly recognised by the major donors and are considered in aid allocations while the ‘forgotten’ marginalised were not considered by the major donors in the allocation and distribution of donor aid.

The mainstream marginalised groups that were mentioned by most donors and implementing organisations included: women, especially pregnant women; children, especially those under the age of five and orphans; sexual minorities; the physically disabled, including people with special needs; island fishermen (fishermen living on islands across Lake Victoria); refugees and host communities in the West Nile region; and residents in the regions of Karamojo and Northern Uganda.

On the other hand, the ‘forgotten’ marginalised groups included: the elderly; physically disabled women; young boys; young girls, especially out-of-school adolescent girls; albinos; child heads of households; street children; sex workers; single women who are divorced or widowed; children living with HIV and AIDS; and ‘uneducated’ women. Most of the participants directly associated the cause of vulnerability for the ‘forgotten’ group to the armed conflict that affected Northern Uganda for about 20 years.

#### B. How are the marginalised and vulnerable individuals and groups identified?

The participants identified a wide range of strategies for identifying the most vulnerable and marginalised individuals and groups, which was an important step towards allocating and distributing aid based on need. The strategies were categorised into three major themes: (i) use of available data and statistics; (ii) consultation and engagement with relevant stakeholders; and (iii) undertaking primary studies.

**i**. **Use of available data and statistics**

Most of the participants across the various participant groups mentioned the use of available data and statistics to inform their decision on the most vulnerable or marginalised individuals and groups. These included official government, national, regional and district data and statistics such as the Uganda Demographic and Health Surveys (UDHS) data and other data and statistics from the Uganda Bureau of Statistics (UBoS)as pointed out by one of the donors:

“…*there are national statistics that are usually updated*, *for example the Uganda Bureau of Statistics usually researches on various aspects*, *and come up with specific indicators that usually include vulnerabilities*, *like for example we would be interested in*. *There is also UDHS surveys*, *Uganda Demographic and Health Surveys have some specific areas of interest…*”

(GI, Donor, 007). Most implementing partners tended to depend more on local district data and regularly engaged with the local district statisticians to get up-to-date data.

Participants highlighted that the key indicators that they focus on in these available data and statistics are poverty and health indices for the regions, districts and counties. Some mentioned that they also make use of other vulnerability indicators that are captured in these data sets. The use of available data and statistics was by far the major strategy pursued by donors and government officials in identifying the most vulnerable/marginalised groups, although they acknowledged that it might not always be up-to-date.

**ii**. **Consultation and engagement with relevant stakeholders**

Another common strategy used by participants in identifying the most vulnerable and marginalised groups was through consultation and engagement with major stakeholders within the donor and recipient landscapes. All participants within the donors and implementing partners’ categories mentioned consultation with government officials at the national, district, county and/or sub-county levels in the process of identifying the most vulnerable/marginalised in the allocation and distributing aid. Consultation and engagement with government officials at the national level was mostly mentioned by participants in the donor group while consultation with government officials at the district level and below was largely mentioned by the implementing organizations.

Most of the implementing partners also highlighted that consultation with the local district officials provided an idea of which other organizations are already working with the identified vulnerable groups and identifying other vulnerable groups that are not currently being covered to avoid duplication as highlighted by an implementing partner participant: “*…I actually got a very good support from them [*local government authorities*] and they were able to guide us by telling us this area is already being taken up by this partner*, *we are locating you to another area*, *where there is a lot of need*, *so we really got good support from them and we are working together with them*.” (II, IP, 001)

In addition to the government officials, donors mentioned the crucial role played by the implementing organizations in providing information on the most vulnerable/marginalised groups due to their strong engagement with the local communities where they distribute the aid they have received. Strong and continuous engagement with local community stakeholders, including community elders, women and youth groups, community volunteers, and potential beneficiary groups was also a major practice mentioned by implementing partners in identifying the most vulnerable groups. Due to their deep understanding the milieu they often use participatory approaches and at time unconventional methods to accomplish this task.

“*I have engaged in community approaches where for instance we would go in the communities*, *the first thing you do is you map out; how do you know the very poor in your community*?, *so when they do all that mapping like I said in the communities*, *they are the ones who would give you that information*. *You say ‘who are the very poor here*?*’*, *then they will say the very poor are people when you go to their homes*, *they have their local indicators you know not accepted by economists*, *so they tell you*, *you see if you go to a household*, *you find grass*, *the compound is not clean and those people are normally very old*, *so when you enter their house*, *they don’t have any stock of food*, *they give the indicators*. *What about the moderates*?, *they will tell you*, *do you know some very poor people*, *do you have these categories of people that you talked about*, *who are they*? *They will tell you*, *they will give you names*. *I told you the whole trick about selection of beneficiaries lies in the community*, *they will always tell you the truth*”

(II, IP, 003)

**iii**. **Undertaking primary studies (needs assessments, feasibility studies and outsourcing to external consultants)**

Most of the participants, especially among the donors and implementing partners also mentioned that one of their preferred strategies to identify the most vulnerable/marginalised groups was to undertake primary studies or commission the task to an independent consultant/firm. The former was more common among the implementing partners while the latter was mentioned mainly by the donors. The various forms of primary studies that participants used to identify the most vulnerable groups include needs assessments/baseline surveys, vulnerability assessments, and outsourcing to external consultants. These methods typically focused on capturing health and demographic, and socio-economic data.

Two implementing partners mentioned undertaking vulnerability assessments using the Uganda vulnerability index that aims at classifying individuals and groups into different categories of vulnerability as pointed in the following quote from a group interview with an implementing organisation: *“*…*on some projects*, *we use the Uganda vulnerability index tool to ascertain the level of vulnerability ranging from generally vulnerable*, *critically vulnerable such that we can ascertain*. *You know most of the time when you are using these tools*, *you will be asked certain questions*, *you may not know why they asked you*, *so when we get back and analyse some of these forms*, *then we get to know exactly how to categorise their vulnerability levels*.” (GI, IP, 002)

The Uganda vulnerability index is a tool that is approved by Uganda’s Ministry of Gender, Labour, and Social Development to identify vulnerable households and the extent of their vulnerability. It was approved for use during the period 2011–2012 and by 2012–2013 up to five U.S government-funded implementing partners in the country had stated using it.

Most of the participants typically used one or more of the strategies described above and tended to prioritise the use of primary and/or secondary data in making such decisions.

### *II*. Strategies for ensuring equity

#### A. What does ‘equity’ entail?

Prior to exploring the strategies employed by the study participants in incorporating equity in the allocation and distribution of development aid, we explored whether there is an intentional effort to focus on equity and their notions of the meaning of the concept of equity. The views expressed by the participants were varied, ranging from ‘equal distribution of aid to targeted groups’ to ‘investing more in disadvantaged groups’.

**i**. **Lowering the gaps in access to services**

Reducing any gaps in access to services between regions, districts and communities was one of the predominant notions of equity mentioned by participants especially those within the donors and government officials’ categories.

“*My understanding of equity is bringing or lowering the gaps in between and promoting similarities in the levels*, *say access to services*, *access to goods*, *access to whatever… At least where there are gaps*, *reducing those gaps*, *bringing those who are down at least to make them accessible to whatever is in question* …”

(II, Donor, 003)

**ii**. **Investing more in disadvantaged populations to address differences in capabilities**

Closely related to the notion of equity as reducing the gaps in access to service was the notion that equity meant ‘investing more in disadvantaged populations’. This could involve building the capacity and capability of the less empowered and that involves a deliberate set of activities.

“*My understanding of equity is that for instance*, *I am standing tall*, *I have capacity okay but how do I help this person to stand tall and have capacity as I do*, *is that equity……I think equity is more of investment in the person who is disadvantaged*, *it is intentional*, *it has to be deliberate*”

(II, Donor, 005)

**iii**. **Inclusion of all segments of the population in the national budget**

One of the government official’s notion of equity was ensuring that the national budgeting process includes a broad spectrum of the various segments that make up the national population. Such an endeavour normally considers children, young people, the elderly, and people with disability as well as regional balance.

“*We are looking at two key things*, *gender and equity and under equity*, *we are looking at the variable of age*, *does somebody consider children*, *issues of youths*, *issues of the elderly because they are ignored*. *Then also under equity we are looking at disability*, *we want people to look at different disabilities among beneficiaries and see how they are budgeted and then lastly under gender*, *we look at geographical inequalities because we know for example like in Uganda*, *we are not at par in all regions*, *some regions have lagged behind for different reasons*, *so we look at if you are having a programme*, *are you having affirmative actions*?”

(II, Gov’t, 003)

**iv**. **Equal distribution of aid among target groups**

Another common notion of equity that was common among the participants was the perception of equity as equal allocation and distribution of aid across the targeted groups, taking into consideration the proportion of the target population across the target communities/areas. This view of equity was more common among the implementing partners as highlighted in the quote that follows: “*We want to have equal distribution although when we talk about equal*, *you know*, *equal cannot be equal in other word but we try to make things more or less to everybody that is within the target group…within our catchment area*, *we also try to distribute at equal levels*. *We give a portion to Gulu*, *we give a portion to Amuru*, *we give a portion to Nowya*, *but also depending on the known target population within area because we may have more people here than in Nowya*, *so we try to calculate the portion and then we give according to that*.” (GI, IP, 007)

#### B. How is equity operationalised in the allocation and distribution of aid?

Participants highlighted a broad range of strategies for ensuring equity in the allocation and distribution of development aid. Most of the strategies identified were linked to their perceptions of the meaning of equity.

**i**. **Focusing on gender, needs, disability, children, youth and perceived vulnerable groups in funding allocation**

The most common strategy mentioned by participants, especially the donors was focusing their development aid towards specific known marginalised/vulnerable groups while paying attention to gender, human rights, environment and needs. This meant that implementing organisations developed potential proposals within these areas of focus. Several donors highlighted that this focus is not only at the level of allocation, but they are also closely monitored during project implementation to ensure that implementing partners adhere to what is written in the funded proposals.

*“*…*in the calls for proposals*, *we make it very clear that the responses and submissions have to be gender sensitive*, *they have to be responsive to the needs of the people*, *persons with disabilities*, *to the needs of the children rights*, *to the vulnerable communities for example… So those indicators are part of the evaluation we look at if they draft them*, *they must address issues of women*, *issues of gender*, *so they form criteria under which we assess those calls*. *So*, *unless the proposal really answers those specific issues*, *it wouldn’t definitely succeed*”

(II, Donor, 002)

**ii**. **Prioritising Northern Uganda and Karamoja in funding allocation**

Another common strategy employed by participants to ensure equity was prioritising the regions of Northern Uganda and Karamoja by donors in their funding allocations, which directly reflects on the preferences of the implementing partners as highlighted by one of the donors: “*We influence their choices*, *sometimes in areas and for example as development partners and democratic governance facilities*, *they are instances where the donor partners emphasise; ‘please look in the Northern Uganda*, *look into the Karamoja regions because you see that is where we need extra push’*…” (II, Donor, 005). Their main reasons provided for prioritising these regions was because of their poor human development indicators and their past conflict history. Many viewed this as a form of compensation for the stagnated rate of development that was engendered by the armed conflict that engulfed the regions.

**iii**. **Targeting both refugees and host populations in refugee-hosting districts**

Three of the donors incorporated equity in their funding allocations by supporting projects that target both refugees and host populations in refugee-hosting districts: “… *I think the refugee approach project is a good example of it*. *You divide the money*, *even though we have the policy of 70–30 [70% of aid on refugees and 30% on host communities]*, *in reality the host communities can get a bit more because they participate in the projects and it makes sense*, *even if it was 50–50*, *it still makes sense* …” (II, Donor, 001).

This strategy was more common among the Nordic donors.

**iv**. **Prioritising the critically vulnerable in any aid distribution process**

Prioritising the level of vulnerability during the distribution of aid was one of the most common strategy mentioned by the implementing partners. This ensured that in any distribution exercise those that are categorised as ‘critically vulnerable’ are prioritised.

“*…we start with the critically vulnerable* [based on the Uganda Vulnerability Index] *and then if we finish and we still have space*, *then we bring the moderately vulnerable households into that until when we meet that target*…”

(GI, IP, 002)

**v**. **Using specific tools and consultants to ensure that major equity issues are addressed in proposals**

The use of tools that specifically capture equity issues and the use of “equity consultants” was also another strategy employed, especially by donors to ensure equity is an integral component of their development aid. The tools constituted an integral part of proposal acceptance, review and monitoring and tend to capture a broad range of equity issues. Some of the donors mentioned that in situations where interesting proposals are received from implementing partners that fail to address some of the equity concerns, they work with them to ensure that those issues are incorporated within the proposal before funding is allocated to the project.

Several donors also mentioned using external consultants to evaluate accepted proposals and monitor funded projects to ensure implementing partners adhere to principles of equity during project design and implementation. This was a more common strategy for funded projects and constituted part of monitoring and evaluation.

“*I will give you an example in this planned XXX* [Name of Donor] *new project*. *That is where we saw the weakness that there is not enough focus on the rights approach and gender and equity*, *so we hired a consultant and she will follow up on that*, *she will go together with the XXX* [Name of Donor] *for monitoring missions like assessing the facilities and look with that [equity] lens and speak to the community … so she can detect weaknesses in the project that can be addressed hopefully when it comes to rights-based approach*, *and when it comes to gender and equity…*”

(II, Donor, 008)

The use of external independent consultants by donors was also acknowledged by a number of implementing partners who have received some of these consultants sent by their donors to work on equity issues.

**vi**. **High level government engagement with donors and implementing partners to incorporate equity in aid allocation and distribution**

All the government officials also highlighted that equity is increasingly a key issue that they engage prospective donors and implementing partners on, including at very senior levels. Although the government officials acknowledged that most donors are increasingly talking about equity with respect to allocating their aid, this is not very evident in their institutional documents. As such, they have been pressing them to consider translating this in their policy documents.

**vii**. **Other strategies**

Many other strategies in ensuring equity were also mentioned by the participants. These included: donors promoting the agenda of mainstreaming gender into all government ministries; funding specific government ministries and departments (eg. Gender, Labour and Social Development) that have a strong equity culture; donors having a specific office/staff responsible for overseeing equity issues; and implementing partners sharing of project selection guidelines with target communities in advance.

### *III*. Challenges to ensuring equity

Participants identified a number of challenges to effectively incorporating equity in the allocation and distribution of development assistance. Most of the challenges were highlighted by the donors.

#### i. Unfamiliarity with and poor understanding of the concept of equity among implementing partners

Many donors hinted that a number of sectors and implementing organizations are unfamiliar with the concept of equity. An example that was mentioned was the infrastructure sector. This unfamiliarity with the concept of equity is reflected in the observation that equity is not deliberately and intentionally incorporated into development projects. Considering that equity is not just a sectoral issue but cuts across all sectors, some donors felt that another related challenge was the absence of a critical mass of committed experts across the various sectors to conceive and implement equity-based policies and practices.

Related to the unfamiliarity of the concept of equity among some implementing partners, a number of donors also raised the issue of poor understanding by some implementing partners and donor colleagues on what equity entails. This is because the concept of equity is quite new for many organisations, with some failing to distinguish equity from equality. With failure to adequately understand what equity entails, this has led to poor application of equity in the allocation and distribution of aid among some development aid stakeholders.

#### ii. Difficulty to operationalise donor’s equity model

Among some donors who had existing institutional models to ensure equity remains an integral component in allocating and distributing their development assistance, some found the models difficult to operationalise. For example, one of the Nordic donor participants found the model overly theoretical in design, lacking a strong operational/practical component, like translating the findings from the model into the process of allocating and distributing aid as exemplified in the following quote: “*We have a whole model on this*, *which is a bit tricky to explain* … *it is actually very theoretical but to be honest*, *I don’t know how we can use it in the programming but that is part of the assignment of midterm review*, *that is where we are*, *we are working on that now*” (II, Donor, 001)

It was therefore understandable among some donors why some of their implementing partners struggle with incorporating equity in the distribution of their aid, since some of the donors also struggle to appropriately convey these issues to them.

#### iii. Political interference

Most partners also highlighted the role of political interference in the allocation and distribution of development aid. This inference came from both politicians in both the donor and recipient countries. Most participants actually acknowledged that donor aid was a political tool, and in some instances, it is allocated and distributed to achieve a particular political objective, which might contravene some basic principles of equity. This was quite common with bilateral aid that is decided at the headquarters with senior level politicians from the donor and recipient countries.

Additionally, some implementing partners also recounted experiences where local politicians and donors have interfered with their selection of beneficiary communities and nature of aid to be delivered, without due consideration to the needs and level of vulnerability of the communities as highlighted in the quote that follows: “*You know*, *sometimes what happens is the donor will tell you; these are the areas that we want to work in and you know there is very little room for flexibility*, *so that is where their problem has been*. *You might know that this is a problem*, *but your donor says we want you to do ABC*…” (II, IP, 008)

#### iv. Existing national data upon which funding decisions are made are not adequately disaggregated

Another major challenge raised by some donors to ensuring equity in the allocation of development aid is the lack of adequately disaggregated data that allows for effective analysis. A case in point is the lack of disaggregation with respect to different population groups and also lack of disaggregated data at the district level.

“*But overall there are only two [*data sources*] that we look at; that is the Demographic and Health Surveys*, *and the annual health sector performance review*, *where you could see the breakdown but you can’t even see it with respect to the different population groups but you can only see per district*. *Still within a district*, *you can also have a lot of inequalities*. *For example*, *now*, *it will be very difficult to follow up the refugees and communities and inequalities in those refugees-receiving areas*…”

(II, Donor, 008)

#### v. Inability to directly work with local community-based organisations

Several donors also acknowledged that community-based organisations were among the best implementing partners to facilitate the process of incorporating equity in the allocation and distribution of development aid as they had excellent knowledge of the situation on the ground within the communities where they operate. However, some were frustrated by the fact that they do not have the mandate to work directly with these small community-based organisations but rather through the relatively bigger civil society actors and other donors.

“…*the problem is that we cannot channel money directly [*to the local community-based organisations*]*, *it has to go through larger organisations or funders*, *something like that because if we would have had bilateral agreements with small organisations*, *it would have been more helpful*, *they are really important*, *we do support them*, *indirectly*. *For us it’s not possible to work directly with community-based organisations*.”

(II, Donor, 001)

#### vi. Corruption

All participants mentioned the issue of corruption as a major challenge to incorporating equity especially in the distribution of development aid. Donors mentioned that this was pervasive both within the government and the implementing partners and largely undermines equity in the sense that resources that are meant for the vulnerable and marginalised get channelled towards personal gains. Additionally, some donors had to stop some programmes because of rampant corruption and mismanagement of development aid by the government and implementing partners.

Some implementing partners also acknowledged that mismanagement and discrimination in the distribution of aid has been observed among some of their implementing partner colleagues, resulting in the syphoning of aid from the most vulnerable groups as highlighted in the quote: “…*when you go down on the ground*, *you find that the most vulnerable people have not been able to benefit from this foreign assistance due to issues of maybe mismanagement or discrimination in some other places*.” (II, IP, 013)

## Discussion

This study sought to explore the extent to which equity is incorporated in the process of allocating and distributing donor aid in Uganda, with a focus on conflict-affected Northern Uganda. Our interviews with donors, implementing organizations/NGOs, and government officials yielded several interesting findings. First, we found that although substantial amount of development assistance has been allocated and distributed towards improving the livelihood of many perceived marginalised and vulnerable groups, a substantial number of other marginalised and vulnerable groups appear to have been ‘forgotten’ in this process. Furthermore, donors, government officials and implementing organisations used a combination of strategies to identify the most vulnerable/marginalised groups, including the use of existing data from various sources, consultation and engagement with relevant stakeholders, and undertaking primary data collection. Additionally, participants expressed a broad range of notions on their perception of what equity entails in the process of allocating and distributing development aid, and a wide range of strategies for incorporating equity in the allocation and distribution of aid. Finally, participants identified substantial number of barriers/challenges in ensuring equity-based allocation and distribution of development aid. To the best of our knowledge, this is the first study in Uganda that has explored the perspectives of key development aid stakeholders in the allocation and distribution of development aid. As such literature on these issues globally and in Uganda is largely unavailable.

Within the context of the SDGs equity has been mentioned as a key ingredient for achieving success as it seeks to ‘leave no one behind’. With many developing countries including Uganda still dependent on development assistance to achieve their national development plans, the important role that donors can play in supporting the attainment of the SDGs cannot be overemphasized. Donors are being encouraged within the SDG agenda to channel more aid to countries where the needs are greatest in line with their national development plans, to contribute towards reducing inequalities within the country. Our study was designed to gauge the extent to which donors and other important development aid stakeholders such as implementing organisations and the government perceive and operationalise this SDG target.

Our findings show that participants had diverse notions of what equity meant for them and their institutions and organisations, with the predominant views associated with the principle of equity as ‘equal concern for people’s needs’, which argues that goods and service should be distributed people proportionally to their level of need and nothing else [8]. Within the context of the SDGs this appears to be the prevailing view of equity as it aligns with the goal of reducing inequalities between and within countries. It was also clear from our findings that many participants equated equity to equality, which should raise a concern as applying principles of equality such as equal allocation or distribution to specific groups, sectors or individuals might not necessary contribute to reduce the gaps between the ‘haves’ and the ‘have nots’. That is precisely one of the major failings in the predecessor of the SDGs, the Millennium Development Goals (MDGs) [18], where although overall gains were made at the national level, regional, groups and other local inequalities were exacerbated in some countries. With diverse understanding of what equity entails, it was therefore not surprising that participants employed a vast array of strategies to incorporate equity in the allocation and distribution of aid, mostly targeting the aid towards perceived marginalised/vulnerable groups. While this might be an easier approach, it may however fail to reach to marginalised/vulnerable groups that are not within the mainstream. The situation may even be dire for individuals and groups with multiple ‘non-mainstream’ vulnerabilities such as disabled out-of-school adolescent girls, adolescent boys living with HIV who are head of households etc. To ensure that equity becomes a reality in the allocation and distribution of aid, it would be important for donors, governments and implementing partners to develop a common understanding of what it entails and how to achieve it within a specific country’s context. Additionally, the government and her development partners need to generate local level disaggregated data that will facilitate the process of identifying the most vulnerable/marginalised groups and such data needs to be updated on a regular basis.

We observed that although most of the mainstream and popularised vulnerable and marginalised groups were considered by donors in the allocation of development aid, some emerging marginalised/vulnerable groups did not appear to be considered in this process. These included a broad range of groups; the elderly, physically disabled women, young boys, young girls (especially out-of-school adolescent girls), albinos, child heads of households, street children, sex workers, single women who are divorced or widowed, children living with HIV and AIDS, and ‘uneducated’ women. These group of ‘*forgotten*’ marginalised/vulnerable groups were mainly provided by local community-based organisations that are not funded by the major international donors. This discrepancy on those who constitute the most vulnerable/marginalised groups between the donors and the implementing partners possibly reflects the challenge that some donors mentioned that their mandate does not permit them to work directly with local community-based organisations although the believe they are the ones that are most knowledgeable of the situation on the ground within the communities. This also raises concerns on the extent to which the bigger civil society organisations and implementing partners that receive aid from the major donors are raising the concerns of these ‘forgotten’ groups from their supporting organisations deep within the communities with the major donors. If bridging inequalities among groups within the country is an important consideration in donor aid allocations, it would be necessary to consider the afore-mentioned groups in future aid allocations.

We found that participants employed a combination of strategies to identify the most marginalised/ vulnerable individuals and groups. It was important to observe that participants generally relied on more than one strategy to identify the most in need often including consultation and engagement with relevant key stakeholders. While the donors undertook their consultations and engagement with key national stakeholders such as government officials and the bigger civil society actors, the implementing partners tended to undertake such activities with stakeholders at the district down to the village level, where the donor-funded projects tended to be implemented. Participants also reported relying massively on data, either freshly collected data or available secondary data. The importance that participants accorded to the role of data in identifying the most vulnerable/marginalised was not particularly surprising although in reality the extent to which this is effectively implemented is difficult to ascertain. This is because a recent study in Uganda found that donor aid tended to not be targeted to localities with the worse socioeconomic conditions [19]. Additionally, empirical studies elsewhere [20–22] have also found that within aid recipient countries, aid disproportionately flows to regions with more richer people, and does not flow to the poor. While our study is unable to effectively assess this in the context of Uganda, anecdotal evidence seems to support this assertion.

Additionally, the role of consultation and engagement with relevant key stakeholders in this process is commendable as it is a critical step towards ensuring transparency and galvanising efforts towards community ownership of potential projects. The use of locally developed tools to assess vulnerability such as the Uganda Vulnerability Index is an interesting observation as in other settings non-locally adapted tools could be utilised which might not be effective. The use of locally developed or adapted and validated tools should therefore be encouraged in undertaking such assessments to ensure a thorough identification exercise. While such assessments might be quite costly for the local community-based organisations, general mapping and assessment of different counties and sub-countries within a district can be undertaken by the bigger civil society organisations or the government and such data made available to donors and implementing organisations when needed.

A recent study [23] found that donors considered a number of factors in the allocation and distribution of their aid to a country, including the health needs, domestic capacity, expected impact, and the cross-cutting concern for equity, in the case of development assistance for health. However, the relative importance of each of these factors varied across donors as well as the indicators used to operationalize each of the factors, a similar situation observed in our study. Across a number of donor participants, it was clear that some of the major funding decisions are made at the central level in their home countries by senior level politicians at the ministry of foreign affairs. However, for donors keen on using their aid to support the recipient countries to attain the SDG of reducing inequalities within the country, it will be important be prioritise the value of equity in such considerations. This should involve strong involvement of relevant stakeholders in the recipient country (e.g. staff in aid recipient country office) as well as clear and transparent policies and guidelines on how such decisions are made. This would also help address the perception among some implementing organisations and government officials that donor aid allocations are driven by other considerations other than equity.

Finally, our study highlights that across our study sites substantial challenges remain in the effective incorporation of equity in aid allocation and distribution. Considering that the concept of equity is relatively new within the context of development aid, we were not surprised that some major challenges were linked to poor understanding of what equity entails, poor familiarity of some sectors and implementing partners to the concept, and difficulty among some donors to operationalise their institutional equity models. With current initiatives within the Ugandan government to promote a system of equity budgeting and mainstreaming of gender within all government ministries, this will go a long way to improve local knowledge and capacity on equity across other sectors and institutions. Furthermore, our findings showed that corruption and mismanagement of donor aid within both the public and private sector seriously undermined initiatives to incorporate equity in aid allocations and distribution as most often funds meant for the most marginalised/vulnerable get siphoned for personal use. Most donors mentioned that because of the precarious situation they had curbed some of their direct funding to the state (budget support) or channelled it through civil society organisations as project aid. Reports of corruption and mismanagement of donor aid in Uganda is not a new phenomenon. In 2012, millions of dollars of aid that was supposed to be used for recovery efforts in conflict-affected Northern Uganda by the Office of the Prime Minister was reported embezzled and diverted to private accounts, resulting to many donors withdrawing their support to the government. For aid to have its intended effect of reaching to the ones most in need, the challenges identified need to be addressed from the sides of the donors, government and implementing partners. Donors and implementing partners need to reassess their commitment towards embracing equity as a priority consideration in their allocations and distribution. The government and implementing partners need to adopt a more transparent and efficient system in the management of development aid.

## Conclusion

This study has explored the extent to which equity is incorporated in the allocation and distribution of development aid in Uganda, especially in the context of the SDGs from the perspectives of donors, government officials and implementing organisations. Our findings raise important policy issues. It is still not clear how various development aid stakeholder understand the concept of equity in the context of allocating and distribution aid. Moving forward it would be important for development aid stakeholders at the national and international level to have a common understanding of what equity entails and how it can be monitored and assessed in the context of aid allocation and distribution. This will not only harmonise efforts towards a common goal of bridging inequalities between and within countries but will also facilitate comparisons between and within countries.

Additionally, with a number of perceived vulnerable/ marginalised groups apparently ‘forgotten’ in the process of aid allocation and distribution, development aid stakeholders in Uganda, especially the major donors may need to adopt a more comprehensive approach to vulnerability/ marginalization assessment with the goal of developing a more comprehensive list of vulnerable/ marginalised groups. This process needs to be transparent to avoid potential misunderstanding that may emanate from the outcome of such assessments and undertaken on a more regularly basis as vulnerability/ marginalization status may change over time.

Furthermore, it remains unclear to what extent donors prioritise equity when allocating their development assistance to recipient countries, and whether their decisions and actions are in any way aimed at contributing towards reducing inequality between and within recipient countries as recommended by the SDGs. To what extent do donors’ other interests within a country overshadow the expectation that their aid allocation should be done with strong equity consideration? To effectively answer this question, there is the need to undertake a more systematic study among donors to understand the decision making process around aid allocation, and how that is translated into actual allocation to the various national stakeholders, and eventual implementation by the national stakeholders such as the government, international NGOs and implementing organizations—the extent to which donors’ aid allocation intentions are reflected in the distribution and implementation of the aid. In the same light, it would be important to assess the extent to which donor policy documents are aligned with their actions on the ground in aid recipient countries. Lastly, although we observed various strategies employed by the various development aid stakeholders, the effectiveness of these approaches deserves a more systematic assessment to better understand their effectiveness in bridging inequalities between and within aid recipient countries. This should lead to the development of evidence-based best practices in incorporating equity within the process of aid allocation and distribution.

## Supporting information

S1 File Additional file 1. Interview guide.
(DOCX)
