# Supplementary material for: Equity in aid allocation and distribution: A qualitative study of key stakeholders in Northern Uganda
Source: PLoS One. 2019 Dec 16;14(12):e0226612. doi: 10.1371/journal.pone.0226612 (PMC6913922; doi:10.1371/journal.pone.0226612)
Supplement: S1 File — Interview guide. (DOCX) [file pone.0226612.s001.docx]

**Interview guide**

**The role of development aid in reducing group inequalities in maternal health care in conflict-affected settings: A qualitative case study of Post-conflict Northern Uganda**

**International donors**

- Brief introduction on the study objective: The role of development aid in bridging group inequalities in maternal health care in conflict-affected settings.
- How will you describe the current state of inequalities (generally and in maternal health care) in Uganda and Northern Uganda? Which groups will you describe as the most privileged in terms of education, income level, access to health facilities, access to land etc? What is your assessment of the most marginalised groups? Has they been any evolution on this in the past decade? What do you think are some of the factors contributing to these inequalities?
- What are the various forms of development assistance (DA) that you provide for recipient countries? (probe on the proportions and examples)
- How do you decide what amount of money to allocate to these various forms of DA?
- What are the factors that you consider when allocating DA to the government? To what extent is inclusiveness (including all groups) in national plans, policies and activities considered in aid allocation to recipient countries? To what extent is that considered for Uganda? Is this something other major donors are also pursuing?
- How do you assess the government’s commitment to inclusiveness in its plans, policies and activities?
- Are they some specific dimensions of inclusiveness that you focus on – political, social, economic, cultural, justice, security etc? (Probe for details for each dimension)
- To what extent do you consider addressing inequalities in the allocation of aid to Uganda? Are there any specific forms of inequalities that are prioritised? (Probe for details – dimensions: education, health care service provision etc.; group identifiers - regional, ethnic, religious etc)
- To what extent do donors like you promote/ consider inclusiveness/equity in the allocation of DA? (probe the various aspect of inclusiveness – political, economic, social, cultural etc)
- How has this affected the government and implementing partners (IPs) policies and practices on inclusiveness/equity in the implementation of DA project? (probe for examples)
- As a donor, what are the challenges you face in ensuring that the government and IPs incorporate principles of inclusiveness/ equity in the implementation of DA? How do you seek to promote the inclusion of equity-based principles in the management of your DA projects? (probe for some examples)
- As a donor, how will you describe the incorporation of equity-based principles in the allocation of DA in your work in the past decade? (probe on specific actions that have been undertaken)
- In the domain of health, are there any specific considerations in ensuring that your health-related DA is allocated and implemented in an equitable manner – ensuring that members from different groups have equal access to health services? (probe for details on specific considerations)
- In your evaluations of DA projects is equity among beneficiary groups something that is considered? If so, how? Has there been any evolution on this in the past decade?
- Can you share some practical steps that you employ in addressing the various forms of inequalities among groups (probe based on dimensions: education, health care service provision etc.; group identifiers - regional, ethnic, religious etc.)
- As a donor, are you exploring any of the following policies with the government with respect to your DA allocation (probe for details):
- investment, credit, extension policies to help offset regional imbalances;
- education and health policies aimed at ensuring ethnic/religious and regional balance in access;
- policies to outlaw discrimination, including fair employment legislation;
- policies to help disadvantaged groups to realise their legal rights, e.g. via legal aid;
- policies towards rights to land and natural resources to ensure balance in access and benefits;
- policies towards achieving equality in cultural recognition;
- policies to regulate the media to ensure equal access; and
- policies towards civil society
- From your experience in allocating DA, to what extent do you think DA can serve as a tool for reducing maternal health care inequalities among groups? What are some potential channels/ mechanisms through which DA can act to reduce maternal health care inequalities? To what extent does bridging maternal health care inequalities among groups within DA recipient communities affect the level of community cohesion?
- What are some models of incorporating equity (especially reducing horizontal inequalities) in the allocation of DA – in conflict-affected and non-conflict affected settings?

**Government Officials and Implementing NGOs**

- Brief introduction on the study objective: The role of development aid in bridging inequalities in maternal health care in conflict-affected settings.
- How will you describe the current state of inequalities in Uganda and Northern Uganda? Which groups will you describe as the most privileged in terms of education, income level, access to health facilities, access to land etc? What is your assessment of the most marginalised groups?
- What are the various forms of DA that you receive from international donors? (Probe for details)
- Is this DA tied to any specific conditions? (probe for details of the conditions)
- How do the key conditions differ from one donor to another (follow-up with the major donors)
- Based on your experience, what are the factors that donors consider in allocating DA? Are these factors the same across the donors?
- Are issues around inclusiveness or reducing inequalities one of the considerations/ conditions from the donor? (probe on the perception and understanding of inequalities)
- In this study, we are particularly interested in how DA can serve as a tool for reducing maternal health care inequalities among groups in conflict-affected settings. To what extent do DA projects seek to achieve this objective? Is this something that is considered in the delivery/implementation of DA projects in conflict-affected communities? (probe for specific experiences)
- If yes, are they some practical steps/ strategies that you incorporate in your work to achieve this? – budget, project etc (probe for concrete strategies)
- Is reducing maternal health inequalities among major groups being monitored in communities receiving DA? If so, has that had some effects in local policies and programming?
- In your experience what are the best forms of DA that can substantially reduce maternal health inequalities among groups? (probe details on the types of DA)
- Within the communities where you deliver DA, are there occasional concerns among different community groups with respect to perceived non-inclusiveness? (Probe for the various types – dimensions: education, health care service provision etc.; group identifiers - regional, ethnic, religious etc.)
- If yes, how have such issues been dealt with by your organization and the local government?
- In the past decade there has been a growing global attention on aid effectiveness. What is your perception of what aid effectiveness is all about? How will you describe DA that is effective? How will you assess aid effectiveness?
- What are the factors that affect the effectiveness of DA? What are the facilitating factors? What are the barriers?
- What are some of the strategies for ensuring that DA is allocated/ delivered in a manner that ensures equal access to all groups and reduces maternal health inequalities among groups?
- From your experience in managing/distributing DA, to what extent do you think DA can serve as a tool for reducing maternal health inequalities among groups? What are some potential channels/ mechanisms through which DA can act to reduce maternal health inequalities among groups? To what extent does bridging maternal health care inequalities among groups within DA recipient communities affect the level of community cohesion?
- What are some models of incorporating equity (especially reducing horizontal inequalities) in the allocation of DA – in conflict-affected and non-conflict affected settings?

**Government only**

As a government, do you consider any of these issues in the distribution/ implementation of DA?

- investment, credit, extension policies to help offset regional imbalances;
- education and health policies aimed at ensuring ethnic/religious and regional balance in access;
- policies to outlaw discrimination, including fair employment legislation;
- policies to help disadvantaged groups to realise their legal rights, e.g. via legal aid;
- policies towards rights to land and natural resources to ensure balance in access and benefits;
- policies towards achieving equality in cultural recognition

**For NGOs only**

In the conflict-affected areas where you have implemented DA projects, how will you describe your assessment of the following situations with respect to the situation on the ground (probe for some examples)?:

- Perceived level of access to and distribution of basic social services – health facilities, food aid, schools, water, electricity etc.
- Perceived differences in the provision/ supply of basic social services to different groups – patronage or nepotism
- Perceived uneven distribution of basic social services within communities – very few services in specific communities
- Perception of political inclusiveness: feelings of political inclusiveness or marginalisation; level of perceived representation in the local government; perceived level of (dis) satisfaction with local political processes and local government; perceived level of participation in local political processes and decision-making; perceived concentration of power in the hands of specific groups;
- Perceived extent of political inclusiveness – civil service, army, police etc.
- Perceived unfair access/ favouritism to employment opportunities, including recruitment process
- Perceived favouritism in the distribution/ allocation of land resources (agricultural land, issuing of land permits); job opportunities

**Women (Women who live in areas where aid projects have been implemented)**

- Brief introduction on the study objective: The role of development aid in bridging inequalities in maternal health care in conflict-affected settings
- How often do you use the local health services in this area? How far are there from the community? Do many women go to seek for these services?
- In this community which category of women do you think suffer the most to get health care? Who are those that you think are privileged in getting health care? (probe exploring identifiers).
- Have you received any assistance from NGOs in the past years? What has been the nature of such assistance? (probe on the NGOs involved and the specific types of assistance)
- Considering all the aid that has been provided in your community, which type do you consider most important for improving the situation of maternal health in the community? (Probe about the various types mentioned, along with examples). Which type of aid do you think helps the most in reducing differences/ improving equity in access and utilisation of maternal health care among groups?
- What is your perception of the effect of the aid? Has it been helpful? What do you like/ dislike most about the manner in which the aid is delivered?
- Have you experienced any situation where someone did not give you or someone aid because he/she belonged to a different group? (probe for details if someone has been given aid due to their group affiliation)
- If yes, what did you do? How was the issue resolved?
- Have you sought healthcare in any local facility? What was your perception of the way you were treated? How will you described your level of satisfaction with the care you received?
- Have you ever felt discriminated against in accessing healthcare as result of the religious or ethnic group you belong to? (probe for details, what was the event, what happened, how did you react, did you report to someone? Was the problem resolved etc?)
- Have you heard of experiences of other women affected by the same situation within this area? Is this a common occurrence in this area?
- How do you perceive the aid that is generally delivered to this community? (Has it helped to better unite people from different groups together? Has it created conflict between some groups at time? Etc.) (probe for specific examples). Has it had any effect on improving access and utilisation of maternal health care among the marginalised/ under-privileged women? (probe for examples and potential mechanisms)
- Having observed DA projects in this community how will you describe your understanding of the following issues (probe for examples where applicable)?:
- level of access to and distribution of basic social services – health facilities, food aid, schools, water, electricity etc.
- differences in the provision/ supply of basic social services to different groups – patronage or nepotism
- distribution of basic social services within communities – very few services in specific communities
- political inclusiveness: feelings of political inclusiveness or marginalisation; level of representation in the local government; level of (dis) satisfaction with local political processes and local government; level of participation in local political processes and decision-making; concentration of power in the hands of specific groups;
- extent of political inclusiveness – civil service, army, police etc.
- unfair access/ favouritism to employment opportunities, including recruitment process
- favouritism in the distribution/ allocation of land resources (agricultural land, issuing of land permits); job opportunities
- To what extent to you believe that the manner in which DA is distributed with a community can improve peaceful co-existed among religious and ethnic groups? How can that process be maximised to improve access to maternal health care and strengthen community cohesion among groups?
- To what extent do you believe that unequal access to healthcare services by women from different groups can undermine community cohesion? How will that be manifested within the community? (probe for examples)
- With respect to the NGOs that visit your communities to provide mobile health services, how do you perceive the manner in which they provide services to the various groups in the community? Do you feel that all the groups are equally treated? Do you feel every women has equal access to these services? How will you describe the attitude of the staff towards women from different groups within the community? Have they been any complains of favouritism towards a particular group?
- What are some of the ways that you think DA can be better delivered in your community to ensure that every woman from different groups benefits? What are the things that make the delivery of these DA projects in your community not accessible to every woman? What do you think the government and NGOs should do to ensure that every woman enjoys the benefits from aid projects?
